# Use of the Long-Term Conditions Questionnaire (LTCQ) for monitoring health-related quality of life in people affected by cognitive impairment including dementia: pilot study in UK memory clinic services

**DOI:** 10.1007/s11136-021-02762-z

**Published:** 2021-02-11

**Authors:** Caroline M. Potter, Michele Peters, Maureen Cundell, Rupert McShane, Ray Fitzpatrick

**Affiliations:** 1 Nuffield Department of Population Health, Health Services Research Unit, University of Oxford, Oxford, UK; 2 Collaboration for Leadership in Applied Health Research and Care (CLAHRC) Oxford, Oxford Health NHS Foundation Trust, Oxford, UK; 3 Oxford Health NHS Foundation Trust, Oxford, UK

**Keywords:** Long-Term Conditions Questionnaire, Cognitive impairment, Dementia, Health-related quality of life, Multimorbidity, Patient-reported outcome measure

## Abstract

**Purpose:**

The aim of this study was to validate the Long-Term Conditions Questionnaire (LTCQ) among patients using memory clinic services in England. LTCQ is a short self-administered measure of ‘living well with long-term conditions’ that has not been previously tested in patients with cognitive impairment.

**Methods:**

The mixed-methods study included cognitive interviews to test the comprehensibility and content validity of LTCQ from the patient’s perspective, followed by a pilot survey to test the measure’s internal consistency, construct validity, structural validity, and responsiveness. Participants were recruited through memory clinics following a diagnosis of mild cognitive impairment or dementia.

**Results:**

Interview respondents (*n* = 12) all found LTCQ’s content relevant, with only minor formatting modifications required. Among survey respondents (*n* = 105), most patients (86%) were able to self-report answers to LTCQ. High multimorbidity among the sample was associated with reduced LTCQ and EQ-5D scores. Internal consistency of LTCQ was high (Cronbach’s α = 0.93), no floor or ceiling effects were observed, and missing data levels were low. Factor analysis results further supported LTCQ’s structural validity, and predicted positive correlation with EQ-5D indicated construct validity. Score changes observed in a four-month follow-up survey (*n* = 61) are suggestive of LTCQ’s responsiveness.

**Conclusion:**

LTCQ is a valid means of assessing health-related quality of life for people living with cognitive impairment (including dementia) in the early period of support following diagnosis. Owing to high levels of multimorbidity in this patient population, LTCQ offers an advantage over dementia-specific measures in capturing the cumulative impact of all LTCs experienced by the patient.

## Plain English summary

Diagnosis of dementia and mild cognitive impairment (MCI) is improving, but people affected by cognitive impairment need ongoing support to maintain a good quality of life. Current health and social care policy states that improved health-related quality of life should be an outcome of effective services following diagnosis, but this is currently not being measured. The Long-Term Condition Questionnaire (LTCQ) is a patient-reported measure of ‘living well with long-term health conditions’ that could be used to monitor how well people are supported following a diagnosis of cognitive impairment. The purpose of this study was to test the use of LTCQ in English memory clinic settings. For the first phase of research, 12 people affected by cognitive impairment completed LTCQ during interviews and gave feedback about their experiences. A modified version of LTCQ was then completed through a survey taken by 105 people with a new diagnosis of MCI or dementia. Survey results showed that LTCQ scores were worse for patients with multiple long-term conditions (including MCI/dementia) and for patients who required more help in completing the survey. But most patients were able to respond to LTCQ questions themselves, to directly report their  health-related quality of life. 61 patients completed LTCQ again 4 months later, and changes in LTCQ scores reflected whether patients’ health had gotten better or worse since diagnosis. We concluded that LTCQ could potentially be used to monitor health-related quality of life following a diagnosis of cognitive impairment and should be tested further among larger numbers of patients.

## Introduction

Enhancing health-related quality of life (HRQoL) for people affected by dementia has been a key focus of national and international policy for more than a decade [1–3]. As numbers of people living with dementia and other forms of cognitive impairment increase [4], defining and delivering person-centred care for this patient population remains a challenge [5]. Earlier work to increase rates of diagnosis in order to accelerate access to appropriate care has been successful [6]. Current priorities include ensuring that people affected by dementia have meaningful care after diagnosis, with appropriate metrics in place for monitoring this [2]. The intention to monitor ‘the effectiveness of post-diagnosis care in sustaining independence and improving [health-related] quality of life’ for people affected by dementia is well established in England, through both the National Health Service (NHS) Outcomes Framework (indicator 2.6ii) and the Adult Social Care Outcomes Framework (indicator 2F); yet metrics for these indicators remain undefined [7, 8].

It is increasingly recognized that ‘living well’ with dementia is reflected in the patient’s perspective on their own health-related quality of life [9]. Current work seeks to identify appropriate HRQoL outcomes for people living with cognitive impairment including dementia [10, 11], with direct input from patients a critical element for success [12]. Monitoring HRQoL in people with cognitive impairment (including dementia) presents unique challenges: the extent to which patients are able to take part in self-administered measures is unclear [13], and the role that informal carers (i.e. family and friends who provide regular support) could play in routine quality-of-life monitoring is not well defined [14].

HRQoL is “generally considered to reflect the impact of disease and treatment on disability and daily functioning; it has also been considered to reflect the impact of perceived health on an individual’s ability to live a fulfilling life” [15]. It is a subjective assessment that reflects personal aspirations and contexts, so it is essential to measure HRQoL from the direct perspectives of patients as far as possible. An important contribution to the field was the development of the DEMQOL suite of measures for individuals with dementia and their carers [16, 17]. However, as DEMQOL is an interviewer-administered (rather than self-administered) measure, its application for ongoing monitoring of outcomes in clinical practice may be limited. The comorbidity of cognitive impairment with other long-term health conditions (LTCs) also potentially limits the use of dementia-specific measures to clearly defined populations, e.g. those participating in clinical trials. In contexts of high multimorbidity [18, 19], a generic HRQoL measure might be preferred for assessing overall outcomes of joined-up services.

The Long-Term Conditions Questionnaire (LTCQ) is a short self-administered measure of the cumulative impact of long-term conditions. It was designed as a holistic measure to capture traditional and non-traditional domains of HRQoL within a general construct of ‘living well with LTCs’, potentially complementing symptom-burden assessments through disease-specific measures. LTCQ was developed through a series of literature reviews, stakeholder and public consultation, qualitative and cognitive interviews with patients, a translatability assessment, and a first validation survey. Results from the stakeholder phase [20] and qualitative interviews [21] demonstrated in-principle support from health professionals and patients for a quality-of-life measure that works across a large range of conditions. Results from cognitive interviews and the translatability assessment [22] indicated that LTCQ captures areas of importance to people with LTCs, and that questionnaire items are comprehensible and meaningful. The 20-item LTCQ captures a broad range of domains (most represented by a single item) including: sense of control, ability to do meaningful activities, safety inside and outside the home, burden of treatments and services, negative experiences including loneliness and stigma, confidence to self-manage LTCs, and ability to live life as one wants. Results from the first validation study among a diverse sample of patients using general practice and social care services in England [23] indicated that the LTCQ’s psychometric properties including internal consistency and test–retest reliability are excellent. Further Rasch analysis [24] confirmed a single scale for the construct ‘living well with LTCs’. Collectively these studies suggest LTCQ’s potential for use in monitoring HRQoL across a wide range of clinical settings. But to date, the LTCQ has not been tested among patients with cognitive impairment, nor has its potential use by informal carers been assessed.

The aim of this study was to validate LTCQ for use among patients with recently diagnosed dementia or mild cognitive impairment. Through this research, we sought to assess the relevance of LTCQ items (content validity) and feasibility of self-completion among the study population; to understand the burden of LTC management experienced by patients referred to memory clinics; and to identify preferred approaches for, and potential barriers to, the use of the LTCQ as an outcome measure for evaluating post-diagnosis memory services.

## Methods

### Participant recruitment and data collection

This instrument validation study had a two-phase mixed-methods design: qualitative research via cognitive interviews with patients (Phase 1), followed by quantitative assessment of questionnaire responses from a larger sample (Phase 2). Participants were recruited following a diagnosis of either mild cognitive impairment (MCI) or dementia, confirmed by medical staff during a first assessment visit to one of 14 memory clinics based within two National Health Service (NHS) Trusts in South East England. During assessment, patients were asked by staff about their willingness to participate in research. Those who met the inclusion criteria (confirmed diagnosis, willing and able to participate, at least 18 years of age, able to communicate in English) were given a study pack that included an invitation letter and participant information sheet. Patients were excluded if they were deemed too unwell or lacking capacity by memory clinic staff (e.g. patients recently in hospital, receiving palliative care, or with severe memory problems), or where memory clinic staff judged that invitation into the study would cause considerable distress. Clinical data including cognitive function scores and individual diagnoses were available to memory clinic staff for assessing participant eligibility, but these data were not provided to the research team.

For Phase 1, cognitive (‘think aloud’) interviews were used to assess the comprehensibility and content of LTCQ from the patient’s perspective [25] (March-Nov 2017). Participants were asked to complete the 20-item LTCQ in the presence of the interviewer and then to discuss their choices and interpretations when selecting their responses to questionnaire items. An interview guide structured discussion around meaning of specific concepts and terms within the items, clarity of the instructions for completing LTCQ, suitability/comprehensiveness of the response options available, whether there were any unclear or inappropriate questions, and whether or not participants would find it useful to complete LTCQ in health or social care settings. Interviews were conducted at the patient’s home and included some open-ended discussion of participants’ experiences of memory problems and any other LTCs. In line with established guidance [25], cognitive interviews were held in two rounds (minimum five participants per round), with review and potential modification of the questionnaire after each round in response to feedback given by participants.

Phase 2 of the study consisted of self-administered surveys returned by post, taken at two time points (Feb-Oct 2018 for Survey 1, June 2018-Feb 2019 for Survey 2). As with Phase 1, participants were recruited through memory clinics using the same inclusion and exclusion criteria outlined above. The study packs included the full survey (Survey 1), which was comprised of the LTCQ (questions 1–20), a comparative generic measure for health-related quality of life (EuroQol five-dimensional descriptive system with visual analogue scale: EQ-5D-5L with EQ VAS, questions 21–26) [26], a comorbidity scale (question 27), and standard demographics questions (questions 28–32). Participants who were willing to take part in a follow-up survey provided their contact details and were sent a shorter questionnaire (Survey 2) four months later. The follow-up survey included LTCQ (questions 1–20), a question on change in health status since the previous survey, a question on health service use in relation to memory problem, and demographic questions (questions 23–25). The target sample size for Phase 2 was based on COSMIN study design guidance for assessing measurement properties of patient-reported outcome instruments [27]. Initially the target was 200 participants (based on a respondent-to-item ratio of 10 for the 20-item LTCQ), but owing to slow recruitment this was revised to a target of 100 participants (respondent-to-item ratio of 5), which still resulted in ‘adequate’ or ‘very good’ quality of study design as per COSMIN guidance for all analyses undertaken.

The lead researcher (CP) maintained contact with memory clinic staff throughout all phases of the study, for ongoing informal feedback on study recruitment and potential barriers/facilitators for routine collection of patient-reported outcomes data.

### Data analysis

Phase 1 qualitative data were analysed using a framework reflecting the interview guide. For each round of cognitive interviews, participant’s comments on each item of the LTCQ and on the broader topics (e.g. clarity of instructions, appropriateness of response options) were collated and discussed among the research team. Comments were categorized to highlight possible amendments needed to questionnaire items: green (no concerns), orange (requires discussion / clarification), or red (problems raised, may require amendment). Cognitive interviews were conducted until no further ‘red’ items were identified, with a record kept of the research team’s response to each query or comment raised by participants.

Phase 2 survey data were entered into SPSS (version 24), a statistical software package. LTCQ items were scored on a scale from 0 (most negative response) to 4 (most positive response). Items 9–15 are negatively phrased and were therefore reverse-scored. Sums of item scores were calculated and recalibrated to give an overall LTCQ score ranging from 0 to 100, with higher scores indicating a better level of ‘living well with LTCs’. Scores were calculated for responses for which at least 18 LTCQ items had been answered. EQ-5D-5L index values were calculated from a value set for England [28]. EQ VAS is reported using a scale of 0 to 100 (with higher scores indicating better HRQoL) and did not require further transformation. To test construct validity, Spearman’s rank correlation coefficient was calculated to test the associations between LTCQ score and EQ-5D-5L index, and LTCQ and EQ VAS scores. As stated in the study protocol, we hypothesized that LTCQ and EQ-5D scores should correlate in the same direction, with at least moderate strength and statistical significance.

For Survey 1, all LTCQ items were examined for missing data and the measure as a whole was examined for floor / ceiling effects (i.e. 15% or more of respondents scoring the lowest / highest possible score) [29]. Internal consistency (i.e. extent to which items correlate with each other, implying a common underlying construct) was assessed with Cronbach’s alpha statistic [30]. Exploratory factor analysis of the 20 LTCQ items was undertaken for comparison with the initial LTCQ validation sample, using parallel analysis [31] to guide retention of factors. The appropriateness of scoring items as a single scale was evaluated through examination of inter-item correlations (acceptable if 0.8 or less) and item-total correlations (acceptable if 0.3 or more) [32]. One-way analysis of variance (ANOVA) was employed to compare distributions of LTCQ scores among sub-groups within the sample (i.e. by demographics, conditions reported, level of help needed to complete the survey).

Survey 2 responses were also analysed for levels of missing data, floor/ceiling effects, and internal reliability. Changes in LTCQ scores were calculated for each respondent between the two survey time points. As preliminary responsiveness analysis, ANOVA was employed to explore score changes in LTCQ score among groups reporting improvement, no change, or decline in global health status across the survey time points. We hypothesized that groups reporting changes in health status between measurement time points should have statistically significant differences in mean LTCQ scores using the paired t-test statistic, with mean LTCQ score increasing for those reporting better health and mean LTCQ score decreasing with those reporting poorer health. Conversely, we hypothesized that those reporting no change in health status would show no statistically significant difference in LTCQ scores via the paired t-test statistic.

## Results

### Phase 1: interviews

Two rounds of cognitive interviews were held with 12 participants (7 in round one and 5 in round two). Nine interviews were held with memory clinic patients who self-completed LTCQ, two with carers who completed LTCQ with the patient present, and one with a carer who completed LTCQ on behalf of a patient who was not present. Patients who self-completed LTCQ took between 5 and 11 min to complete the questionnaire, mirroring completion times observed during initial development of LTCQ. No suggestions for changes to the content of the questionnaire were made during the first round of interviews. Following patient feedback from the second round, the format of items 13 and 14 was revised to emphasize that these questions included a ‘not applicable’ response option.

Initially a separate proxy version of LTCQ was trialled with carers, which only differed from the original LTCQ in referring to ‘them/their’ rather than ‘you/your’ health conditions. However, it was observed in interviews that patients’ levels of independence in completing the questionnaire varied and would be difficult for memory clinic staff to anticipate in advance. Three patients who completed LTCQ independently nonetheless expressed preference for support from their carers in filling out the form. Two carers noted that they or another family member usually helped the patient with reading and interpreting written correspondence, and that this practice would also be followed in completing a questionnaire. The level of support ranged from reading questions aloud and prompting patients to respond, to more significant help with interpretation of the items. Owing to the uncertainty of the level of support that might be needed, instructions for completing LTCQ were revised to include the possibility of support or completion by proxy. A separate proxy version of LTCQ was therefore not needed, and only the original LTCQ (with revised instructions and an additional item for specifying the level of independence in completing the questionnaire) was used in Phase 2 of the study.

### Phase 2: baseline and follow-up surveys

For Survey 1, a response rate of 26% (107/410) was achieved. Two substantially incomplete responses were excluded, for an analytical sample of *n* = 105. Most respondents (*n* = 85, 81%) consented to completion of Survey 2. Eighty respondents were sent the follow-up survey (aligning with the pre-set end-of-study date), with a higher response rate for Survey 2 (*n* = 61, 76%).

Table 1 shows how LTCQ scores varied by sub-groups within the sample. Respondents to Survey 1 were balanced by gender, with a mean age of 79 years (range 58–91), and 78% reported having at least one other LTC in addition to MCI/dementia. No statistically significant differences were found in LTCQ score distribution by gender, age, marital status, or ethnicity. Statistically significant differences in LTCQ scores were found according to independence in completing the questionnaire (F (2,99) = 3.70, *p* = 0.03); those who reported self-completion (*n* = 45, 43%) had a mean LTCQ score of 76.6, compared to 67.5 for those who had help in completing the form (*n* = 45, 43%) and 64.7 for responses completed by proxy (*n* = 13, 12%). LTCQ scores also varied at statistically significant levels according to multimorbidity; those reporting no additional conditions beyond MCI/dementia had a mean LTCQ score of 74.6, compared to a mean LTCQ score of 59.2 for those reporting 4 or more comorbidities (F (3100) = 4.76, *p* = 0.004).

**Table 1:** LTCQ scores by participant characteristics

Total sample	Mean LTCQ				One-way ANOVA
	N (%)	Score	SD	SE	
	105	71.0	18.9	1.8	
Gender					
Female	51 (49%)	71.8	19.1	2.7	F (1,99) = 0.23, *p* = 0.63
Male	51 (49%)	70.0	18.8	2.6	
* Missing*	*3 (3%)*				
Age					
Under 65	2 (2%)	52.6	1.9	1.3	F (3,95) = 1.96, *p* = 0.13
65–74	28 (27%)	77.5	17.6	3.3	
75–84	48 (46%)	69.6	19.2	2.8	
85 +	22 (21%)	70.0	17.2	3.7	
* Missing*	*5 (5%)*				
Marital status					
Married / partnership	67 (64%)	74.4	17.8	2.2	F (2,97) = 2.12, *p* = 0.13
Widowed	24 (23%)	66.1	17.5	3.6	
Separated / divorced / single	10 (10%)	67.6	22.6	7.2	
*missing*	*4 (4%)*				
Ethnicity					
White British	95 (90%)	71.5	18.7	1.9	F (2,97) = 0.29, *p* = 0.75
Other White	3 (3%)	67.5	22.9	13.2	
Black or Mixed	3 (3%)	78.8	16.3	9.4	
* Missing*	*4 (4%)*				
Help completing questionnaire					
Self-completed	45 (43%)	76.7	17.7	2.7	F (2,99) = 3.70, p = 0.03*
Help with form	45 (43%)	67.5	19.3	2.9	
Proxy completion	13 (12%)	64.7	17.1	4.7	
* missing*	*2 (2%)*				
Co-morbidities					
No co-morbidities reported	23 (22%)	74.6	19.4	4.0	F (3,100) = 4.76, p = 0.004**
1 co-morbidity	40 (38%)	77.0	18.3	2.9	
2–3 co-morbidities	29 (28%)	64.6	15.5	2.9	
4 or more co-morbidities	13 (12%)	59.2	19.3	5.6	

Statistically significant ANOVA results are highlighted in bold

*LTCQ* Long-Term Conditions Questionnaire, *SD* standard deviation, *SE* standard error of the mean, *ANOVA* analysis of variance

Table 2 provides a more detailed comparison of self-reported HRQoL according to condition. Hypertension was the most commonly reported comorbidity (31% of the sample), followed by arthritis (28%), depression (20%), heart disease (18%), and diabetes (15%). Participants reporting no conditions beyond MCI/dementia had higher HRQoL scores for all three measures (LTCQ, EQ-5D-5L index, EQ VAS) compared to those reporting any other condition. As observed in the initial validation sample, LTCQ scores correlated strongly and positively with EQ-5D-5L index values (*r*_s_ = 0.79, *p* < 0.001) and with EQ VAS scores (r_s_ = 0.67, *p* < 0.001).

**Table 2:** Health-related quality-of-life outcomes by comorbidity with MCI / dementia

Health condition in addition to MCI / dementia	N (%)*	Mean LTCQ score (SD, SE)	Mean EQ-5D-5L index value (SD, SE)	Mean EQ VAS score (SD, SE)
No comorbidities reported	23 (22%)	74.6 (19.4, 4.0)	0.85 (0.17, 0.04)	78.9 (13.9, 2.9)
Hypertension	33 (31%)	67.8 (19.9, 3.5)	0.74 (0.22, 0.04)	72.2 (18.5, 3.4)
Arthritis	29 (28%)	70.5 (17.6, 3.3)	0.74 (0.23, 0.04)	73.3 (18.2, 3.4)
*Depression*	21 (20%)	59.4 (14.8, 3.3)	0.64 (0.22, 0.05)	61.4 (20.0, 4.6)
Heart disease	19 (18%)	68.6 (20.7, 4.9)	0.74 (0.22, 0.05)	72.3 (19.3, 4.6)
Diabetes	16 (15%)	66.7 (20.4, 5.3)	0.75 (0.24, 0.06)	69.1 (21.5, 5.8)
Leg pain from poor circulation	15 (14%)	64.2 (17.8, 4.6)	0.68 (0.26, 0.07)	71 (22.0, 6.1)
*Neurological (Parkinson’s, MS)*	11 (10%)	58.1 (16.8, 5.1)	0.60 (0.28, 0.09)	67.5 (16.9, 5.3)
*Lung disease*	11 (10%)	56.1 (15.2, 4.6)	0.55 (0.31, 0.10)	61.5 (18.4, 5.8)
*Cancer in the last 5 years*	8 (8%)	55.2 (22.4, 7.9)	0.64 (0.32, 0.12)	55 (24.3, 9.9)
*Kidney disease*	5 (5%)	57.8 (24.0, 10.7)	0.62 (0.39, 0.20)	58.3 (30.3, 15.2)
*Stroke*	5 (5%)	52.9 (17.3, 7.8)	0.76 (0.13, 0.06)	55.0 (5.8, 2.9)
*Liver disease*	2 (2%)	44.2 (15.3, 10.8)	0.74 (1 response)	60 (1 response)
Total sample	105 (100%)	71.0 (18.9, 1.8)	0.78 (0.22, 0.02)	73.2 (17.7, 1.8)

*LTCQ* Long-Term Conditions Questionnaire, *EQ-5D-5L* EuroQol five-dimensional five-level index value, *EQ VAS* EuroQol Visual Analog Scale, *SD* standard deviation, *SE* standard error of the mean

*Numbers add to greater than 100% because 40% of the sample reported multiple comorbidities

**Fig. 1:**
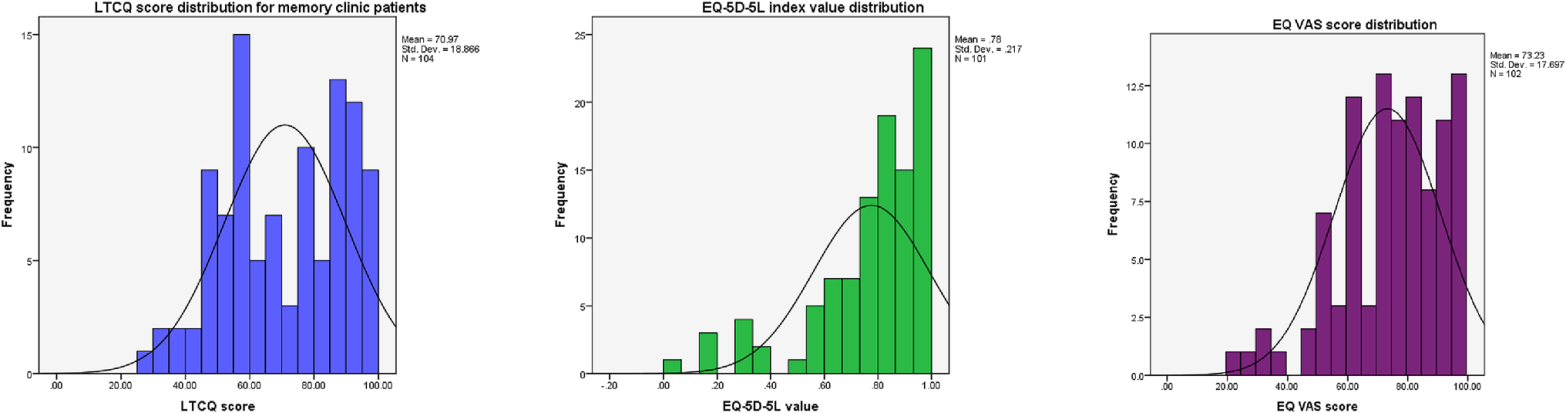
Score distributions for LTCQ, EQ-5D-5L, and EQ VAS for Survey 1 (*n* = 105)

An LTCQ score could be calculated for 99% of the sample, with just one respondent completing less than 18 items. LTCQ scores ranged from 26.3 to 100, with only two respondents scoring the maximum possible score. Therefore, no floor or ceiling effects were observed. Figure 1 illustrates the distribution of LTCQ scores across the sample. While strongly correlated with both EQ-5D-5L index values and EQ VAS scores, LTCQ responses were less skewed towards the most positive scores than either EQ-5D measure. Peaks of LTCQ score frequency occurred in the ranges of 55–60 and 85–90, suggesting a possible bimodal distribution among this memory clinic sample.

Table 3 shows item-level results from missing data and reliability analyses, and exploratory factor analysis. Missing data levels were low (range 0%–5%) for all items. Cronbach’s alpha for respondents completing all 20 items (*n* = 89, 85% of sample) was α = 0.93. This high internal consistency mirrors that observed for the initial LTCQ validation sample. All item-total correlations were acceptable, ranging from *r* = 0.36 (item 12, stigma) to *r* = 0.80 (item 2, ability to fulfil responsibilities). Strong correlations (above 0.7) were observed between the first five items (coping, fulfilling responsibilities, being physically active, feeling in control, doing enjoyable activities) and item 19 (feeling confident to manage health conditions). This clustering of items, reflecting various aspects of autonomy, was also observed in the initial validation sample [23] and through subsequent Rasch analysis [24].

**Table 3:** Missing data and factor loadings for LTCQ items, Survey 1 (*n* = 105)

Item	Missing data	Two-factor solution Pattern matrix^a,b^		Two-factor solution Structure matrix^a,b^		One-factor solution^a^	Corrected item-total correlations (20 items)
		Factor 1	Factor 2	Factor 1	Factor 2	Factor 1	
Felt that your home is suitable for your needs (item 6)	0%	0.87		0.73		0.63	0.60
Felt safe outside the home (item 8)	2%	0.75		0.74	0.34	0.70	0.72
Able to fulfil responsibilities (item 2)	1%	0.75		0.83	0.54	0.85	0.80
Felt confident in managing your health conditions (item 19)	2%	0.73		0.79	0.48	0.79	0.73
Felt in control of daily life (item 4)	2%	0.71		0.78	0.50	0.79	0.73
Had enough support to cope well with health conditions (item 18)	1%	0.70		0.68	0.31	0.65	0.60
Had enough social contact with people (item 17)	3%	0.67		0.69	0.37	0.68	0.65
Able to take part in activities you enjoy (item 5)	0%	0.65		0.74	0.49	0.75	0.74
Felt safe at home (item 7)	0%	0.60		0.51		0.45	0.47
Able to live your life as you want (item 20)	1%	0.57	0.32	0.73	0.60	0.78	0.74
Able to be as physically active as you wanted (item 3)	0%	0.57	0.36	0.74	0.64	0.80	0.76
Able to cope well with health conditions (item 1)	0%	0.56		0.68	0.52	0.71	0.65
*Felt that your health conditions made you unhappy (item 15)	1%	0.49		0.60	0.47	0.63	0.59
*Found health/other services difficult to cope with (item 13)	5%	0.44		0.47		0.48	0.48
Felt you knew enough about your health conditions (item 16)	2%	0.36		0.45	0.36	0.48	0.45
*Felt bothered by symptoms (item 9)	1%		0.65		0.63	0.40	0.39
*Felt lonely due to health conditions (item 11)	0%		0.61	0.36	0.64	0.47	0.47
*Felt more dependent on others than you wanted (item 10)	0%		0.54	0.36	0.59	0.46	0.47
*Worried about being treated differently (item 12)	0%		0.34	0.39	0.45	0.45	0.36
*Found treatments difficult to cope with (item 14)	5%		0.32	0.45	0.47	0.50	0.47

*Reverse-scored item

^a^Extraction Method: Principal Axis Factoring

^b^Rotation Method: Oblimin with Kaiser Normalization

For exploratory factor analysis, parallel analysis [31] indicated initial retention of two factors after extraction through Principle Axis Factoring (PAF). Oblimin rotation with Kaiser Normalization yielded the clearest distinction of factors with minimal cross-loading (see Table 3). The two-factor solution explained 51% of total variance across LTCQ item responses, 43% for factor 1 (eigenvalue = 8.7) and a further 8% for factor 2 (eigenvalue = 1.6). On examination of factor loadings, we noted that these were relatively weaker for factor 2, which included five of the seven negatively phrased items. Previous work has cautioned against misinterpretation of separate factors based on groupings between positively and negatively phrased items [33]. We thus re-analysed the scale though a single factor solution, as indicated by results from the initial LTCQ validation sample [23, 24]. All items contributed to the single factor solution with a minimum factor loading of 0.40. These results mirrored the corrected item-totals from the reliability analysis, further supporting the interpretation of LTCQ as a single scale with a general underlying construct of ‘living well with long-term conditions.’

Table 4 shows LTCQ scores and self-reported change in health status for participants who completed Survey 2 (*n* = 61), four months after baseline. As for Survey 1, missing data across all items ranged from 0 to 5%. Scores could be calculated for 95% of the sample who completed 18 or more items, with 89% of the sample completing LTCQ in full. Internal consistency for the measure remained high (Cronbach’s α = 0.91). LTCQ score at four-month follow-up ranged from 30 to 98.8, so again no floor or ceiling effects were observed. Those who reported better health than at baseline (*n* = 11, 18%) had a mean increase in LTCQ score of 8.4, while those who reported worse health than at baseline (*n* = 13, 21%) had a mean decrease in LTCQ score of 7.6. This result was statistically significant (F(2,54) = 4.63, *p* = 0.01) and is suggestive of LTCQ’s responsiveness to change, although it must be interpreted cautiously owing to the small sample size.

**Table 4:** Change in LTCQ score and global change in health status over four months, Survey 2

Health at Survey 2 compared to Survey 1	N	Mean change in LTCQ score	Mean LTCQ score–Survey 1	Mean LTCQ score–Survey 2	SD	SE	EQ-5D-5L (from Survey 1)	EQ VAS (from Survey 1)
Better than 4 months ago	11	8.39 (n.s., *p* = 0.132)	68.0	76.4	15.3	4.6	0.85	80.0
About the same as 4 months ago	36	− 1.69 (n.s., p = 0.440)	79.2	77.1	17.4	3.0	0.83	77.5
Worse than 4 months ago	13	− 7.57** (p = 0.006)	69.3	61.0	15.5	4.3	0.69	71.3
Total	60	− 0.98 (n.s., p = 0.587)	75.1	73.4	17.6	2.3	0.80	76.6
One-way ANOVA		**F(2,54) = 4.63,** ***p*** **= 0.01**	F(2,56) = 2.44, *p* = 0.10	**F(2,55) = 4.62, *p*** **= 0.01**			F(2,54) = 2.23, *p* = 0.11	F(2,55) = 0.90, *p* = 0.41

Statistically significant ANOVA results are highlighted in bold

*LTCQ* Long-Term Conditions Questionnaire, *SD* standard deviation of LTCQ score, Survey 2 *SE* standard error of the mean for LTCQ score, Survey 2, *ANOVA* analysis of variance

** Statistically significant difference for paired t-test statistic (*p* < 0.01)

*n.s.* ,no statistically significant difference for paired t-test statistic (*p* > 0.05)

## Discussion

This study was the first to test use of the Long-Term Conditions Questionnaire within a clinical population of people affected by cognitive impairment including dementia. It served as further validation of LTCQ beyond its initial sample, which included people living with a wide range of LTCs, e.g. cancer, chronic back pain, chronic obstructive pulmonary disease, depression, diabetes, ischemic heart disease, irritable bowel syndrome, schizophrenia, multiple sclerosis, osteoarthritis, and stroke. Consistency of results from the current and earlier study [23] suggests that the range of LTCQ scores observed is potentially generalizable to the wider population of people living with LTCs, including those with cognitive impairment. It was noted that among this clinical sample a peak in frequency occurred in the LTCQ score range 55–60, which was also observed among a sub-sample of social care service users in the initial validation study but which was not observed among the initial validation sample as a whole. This suggests that patients diagnosed with cognitive impairment may already experience reduced health-related quality of life and may require higher levels of support from services at the point of diagnosis, in comparison to patients diagnosed with other LTCs.

A striking finding was the high burden of multimorbidity within the sample. Those with at least one LTC in addition to MCI/dementia reported reduced HRQoL across all measures, with the effect more pronounced as the number of comorbidities increased. The interplay between cognitive impairment and other LTCs was observed during a patient interview in which the difficulty of managing diabetes was highlighted as the main impact of cognitive impairment; the interview ended prematurely as the patient required help from their carer to address uncontrolled insulin levels after feeling unwell. While condition-specific measures are valuable for their sensitivity in detecting changes associated with that condition (and thus may be preferred in clinical trials for testing efficacy of treatments targeted towards specific symptom reduction) [34], in daily life patients manage the cumulative impacts of all their LTCs. As a more holistic measure that captures the impact of multimorbidity, LTCQ could play a key role in monitoring longer-term changes in HRQoL, which will be impacted by the experience of cognitive impairment and its effects on the management of other long-term conditions.

This study highlighted the capability of many patients with cognitive impairment to self-report their own health-related quality of life. Most patients who took part in interviews were able to complete the questionnaire independently and could engage with the researcher on the relevance of LTCQ’s content for their personal circumstances. When prompted to reflect on whether or not completing LTCQ would be useful within health and social care settings, interview participants saw its value as a tool for reflection on their current health status – but only if completion of the questionnaire resulted in dialogue with health professionals. Several patients anticipated that progression of cognitive impairment would affect their answers to LTCQ, which could be used to track changes in their HRQoL over time. Longitudinal collection of LTCQ data could potentially highlight new support needs from services (both within and outside of memory clinics) as the cumulative impacts of LTCs change.

Nonetheless, high variation of cognitive function among this patient population presents a specific challenge for routine collection of patient-reported outcomes. While the majority of memory clinic patients in this study (10 of 12 interview participants, 86% of survey respondents) were able to give their own answers to LTCQ items, many had some level of help in completing the questionnaire, e.g. help from a carer to stay focused (observed in two interviews) or physical help in filling out the form (reported by 43% of survey respondents). In two interviews and 12% of survey respondents, a carer fully answered LTCQ items on the patient’s behalf. The difficulty of accurately anticipating the level of help needed was noted by memory clinic staff during the first round of interviews, when trying to decide whether participants should be given the self-complete or proxy version of the questionnaire. In the interviews, it was observed that help would likely be given by a carer if this was the usual dynamic in the relationship (e.g. if the carer habitually handled the patient’s written correspondence, or took active part in the patient’s medical consultations). A recognized issue in proxy-reported outcomes is that carers may perceive and report patients’ health-related quality of life differently than patients themselves [35]. This potential limitation in interpretation must be balanced by the need to collect patient-reported outcomes as far as possible for people with cognitive impairment, particularly for ‘seldom heard’ groups for whom HRQoL assessment is often absent from consideration of their service support needs [36].

The small sample size is a limitation of the study. While meeting the ‘very good’ quality threshold for internal consistency and construct validity analyses, with ‘adequate’ sample sizes for factor analysis and responsiveness [27], the study team initially aimed for a larger sample that would have enabled more detailed sub-group analysis (e.g. by specific comorbidities) and more thorough testing of responsiveness. As the first study collecting longitudinal LTCQ data, the follow-up survey results are encouraging: LTCQ scores changed as predicted in the same direction as self-reported change in health status, with a statistically significant decrease in LTCQ scores for participants reporting ‘worse health than four months ago’. However, as the sample size for Survey 2 is only of ‘adequate’ (rather than ‘very good’) quality for responsiveness analysis [27], with change scores for those reporting better health not reaching statistical significance, results should be verified in larger samples.

The sample size reflects modest response to the initial study invitation at clinics, which limits generalizability of the findings. We were somewhat surprised to observe that mean scores for both LTCQ and EQ-5D were higher for this sample than for the initial validation sample, which excluded patients with cognitive impairment [23]. This observation, coupled with slower-than-anticipated participant recruitment, suggests a potential bias in the study results towards the most well patients within the target clinical population. LTCQ’s validity among patients whose cognitive impairment is more severe is therefore uncertain and needs to be tested further in studies for which linked data on individuals’ diagnosis and level of cognitive function are available. In seeking to explain the difficulty of recruitment to the study, we noted that in Phase 1 interviews participants spoke of the diagnosis period as potentially overwhelming, both emotionally and in terms of the amount of information received. For routine collection of patient-reported outcomes we recommend that baseline data are gathered prior to the memory clinic assessment appointment, and/or at a first follow-up assessment to allow for a period of adjustment to the diagnosis.

## Conclusions

The Long-Term Conditions Questionnaire is a valid means of assessing health-related quality of life for people living with cognitive impairment (including dementia) in the early period of support following diagnosis. Owing to high levels of multimorbidity in this patient population, LTCQ offers an advantage over dementia-specific measures in capturing the cumulative impact of all LTCs experienced by the patient. Initial results suggest that LTCQ captures changes in HRQoL as a patient’s health improves or deteriorates over time, but larger-scale studies with linked data on severity of cognitive impairment are needed to further test LTCQ’s responsiveness and use in longitudinal outcomes assessment. It is essential that people with cognitive impairment are given the opportunity to report, as far as possible, on if the health and care services they use enable them to maintain or improve their health-related quality of life. Validation of LTCQ within this patient population increases its utility as a tool for person-centred care across the full range of long-term conditions.

## Data Availability

Data are held by the research team at the University of Oxford, as stated on the Participant Information Sheet: ‘We will keep research data (questionnaire responses) for 10 years and then they will be destroyed. All data use is strictly within the terms of the Data Protection Act (DPA 1998). Only the research team will have regular access to research data, and any data that are shared with others will have names and any other personal information removed so that you cannot be identified from it. Any information collected as part of the study may also be seen by responsible persons from the study sponsor (University of Oxford) or regulatory authorities, but only where it is required for monitoring research. Data will not be shared outside the research team for any other purposes.’
